# Epicardial Fat Thickness in Children with Classic Congenital Adrenal Hyperplasia

**DOI:** 10.4274/jcrpe.galenos.2018.2018.0153

**Published:** 2019-02-20

**Authors:** Kotb Abbass Metwalley, Hekma Saad Farghaly, Abdelrahman Abdelhamid

**Affiliations:** 1 Assiut University Faculty of Medicine, Department of Pediatrics, Assiut, Egypt; 2 South Valley University Qena Faculty of Medicine, Department of Clinical Pathology, Qena, Egypt

**Keywords:** Diastolic function, echocardiography, epicardial fat thickness, left ventricular function, left ventricular mass index, congenital adrenal hyperplasia, carotid intima media thickness, mitral deceleration time

## Abstract

**Objective:**

Epicardial fat thickness (EFT) is an emerging cardio-metabolic risk factor and has been shown to be related to atherosclerosis. EFT has not been studied in the context of CAH. This study aimed to evaluate EFT in children with CAH and its relation to carotid artery intima-media thickness (CA-IMT) and left ventricular (LV) functions.

**Methods:**

Thirty-six children with classical CAH were compared with 36 healthy controls. All patients had confirmed CAH and were receiving steroid substitution therapy. Patients and controls underwent anthropometric evaluation, measurement of fasting lipids, glucose, insulin, homeostasis model assessment for insulin resistance (HOMA-IR). LV functions and EFT were assessed using conventional echocardiography. Duplex ultrasonography was used to measure CA-IMT.

**Results:**

Compared to controls, patients had greater EFT (p=0.001), CA-IMT (p=0.01), LV mass index (LVMI) (p=0.001) and prolonged mitral deceleration time (DcT) (p=0.01). CAH patients also had significantly worse HOMA-IR (p=0.001) than controls. Abnormalities were worse in uncontrolled CAH on treatment. Multivariate analysis in CAH subjects showed EFT correlated positively with waist circumference odds ratio (OR) [OR=1.9; 95% confidence interval (CI): 1.07-1.14; p=0.01], 17-hydroxyprogesterone [OR=1.6; 95% CI: 1.33-2.89; p=0.05], testosterone concentration (OR=1.7; 95% CI: 1.55-2.13; p=0.01), LVMI (OR=1.14; 95% Cl: 1.08-1.13; p=0.01), mitral DcT (OR=2.25; 95% CI: 1.15-2.05; p=0.01) and CA-IMT (OR=1.6; 95% CI: 1.15-2.05; p=0.01).

**Conclusion:**

EFT is elevated in children with classical CAH, particularly in those with poor control, and is correlated with CA-IMT, LV mass and mitral DcT. Measurement of EFT in CAH children may help to identify those at high risk of developing LV dysfunction and subclinical atherosclerosis.

**What is already known on this topic?**

There is an increased risk for cardiac abnormalities in children with congenital adrenal hyperplasia. Epicardial fat thickness is an emerging cardio-metabolic risk factor and has been shown to be related to atherosclerosis.

**What this study adds?**

To our knowledge, this is the first study assessing epicardial fat thickness (EFT) in children with congenital adrenal hyperplasia (CAH). EFT is higher in children with CAH than in healthy children and correlated with carotid intima media thickness, left ventricular mass and mitral deceleration time. EFT may be used as a possible marker of early atherosclerosis and myocardial function in children with CAH.

## Introduction

Congenital adrenal hyperplasia (CAH) is an autosomal recessive condition resulting from mutations in enzymes required for adrenal steroid synthesis (1). Defects in the enzyme 21-hydroxylase, leading to enzyme deficiency, are responsible for approximately 95% of cases (2). CAH is commonly divided into the severe classical and the milder nonclassical form. Classical CAH is generally subdivided, depending on the extent of enzymatic impairment, into the salt-wasting (SW) form, presenting with both cortisol and aldosterone deficiency and the simple virilizing (SV) form, characterized by an isolated cortisol deficiency. Both conditions are associated with androgen excess resulting in virilization of female external genitalia (3). Researchers have long thought that patients with 21-hydroxylase deficiency are at increased risk for cardiovascular diseases due to the resulting high plasma levels of androgens and/or the harmful effects of glucocorticoid and mineralocorticoid treatment (4). 21-hydroxylase deficiency may also have detrimental effects on vascular structures as well as ventricular systolic and diastolic function (5). Obesity, hypertension, dyslipidaemia and insulin resistance (IR) have been found to be associated with both CAH itself and the treatment strategies (6).

Few studies have utilized carotid artery intima-media thickness (CA-IMT) to assess vascular structural changes in children with CAH (4,5,7). A hindrance to the wider use of CA-IMT measurements in the pediatric population is the lack of standardization of CA-IMT values in this age group (8). Epicardial fat thickness (EFT) is a layer of adipose tissue surrounding the heart and coronary vessels which can be measured by ultrasound, a simple, noninvasive procedure (9). EFT is a reliable and sensitive marker of cardiovascular risk and has become an emerging target for therapeutic and medical interventions (10). We are not aware of any published data on EFT in children with CAH. 

The aim of this study was to evaluate the EFT measurement and its relation to CA-IMT and left ventricular function in a cohort of children with classical CAH.

## Methods

### Patients and Methods

This cross-sectional, controlled study included 36 children (11 males and 25 females; mean ± standard deviation age=13.7±2.4 years) with a confirmed diagnosis of classic CAH (4). Diagnosis was made based on clinical signs and biochemical assessment [elevated adrenocorticotropic hormone (ACTH), 17-hydroxyprogesterone (17-OHP), androstenedione and testosterone, in addition to low cortisol]. SW was diagnosed in patients with frank hyponatraemia and hyperkalaemia accompanied by low plasma aldosterone and elevated rennin concentrations (11). Patients were included if they were on glucocorticoid therapy for a minimum of five years. They were recruited during the period between January and December 2017 from the Pediatric Endocrinology Unit of Assiut University Children Hospital, Assiut, Egypt. Patients who had chest deformities, chronic lung disease, poor echo window, pericardial and/or pleural effusion on transthoracic echocardiography were excluded. Thirty six healthy children (7 males and 27 females) matched for age, gender, pubertal status and socioeconomic status were recruited as control subjects from the General Pediatric Outpatient Clinic of the same hospital. None of the controls were hypertensive and none were smokers, on any medication, or had a chronic illness. Controls were attending the outpatient clinic either because of minor illness or accompanying their sick siblings. All patients had classical CAH with 21-hydroxylase deficiency (SW n=30; SV n=6) and were receiving glucocorticoid substitution therapy with hydrocortisone (HC) (n=30) or prednisone (PR) (n=6). PR dose was converted to HC using the conversion assumption that 20 mg of HC is equivalent to 5 mg of PR (12). SW patients were also on 9-alphafludrocortisone therapy at a dose of 50-100 ug/m2/day. The adequacy of steroid therapy was monitored periodically during follow-up visits every 3-6 months by clinical parameters, such as signs of androgen excess, growth curves, bone age and hormonal assay (13). Patients were divided into two groups according to the degree of control on medical treatment, that is those patients with acceptable disease control and children with poor disease control, based on the previously mentioned data (14).

The study protocol was approved by the Local Ethics Committee of Assiut University Children Hospital, Assiut, Egypt (approval number: 312/2017) and also by the Ethics Committee of the Faculty of Medicine, in accordance to the Declaration of Helsinki. Written informed consent was obtained from the parents of all participants.

All patients and controls were subjected to a full medical history-taking as well as a thorough clinical examination. Demographic and clinical data included age, gender, duration of treatment, type and dose of steroids, blood pressure (BP), height, weight and body mass index (BMI). Systolic BP (SBP) and diastolic BP (DBP) were measured in all subjects in the right arm with a standard sphygmomanometer (Exacta) by the same operator. Height and weight were measured using a wall-mounted stadiometer (seca 213 l) and a calibrated weight scale (Uline Industrial Platform Scales), with the child wearing underwear only. BMI was calculated using the following formula: BMI=weight (kg)/height (m)^2^. BMI was expressed as standard deviation scores (SDS) using the Egyptian Growth Reference Data (15). Waist circumference was measured at the midpoint between the lower edge of the ribs in the midaxillary line and the top of the iliac crest by the same clinician. Waist-to-height ratio was then calculated as an index of visceral adiposity. Pubertal status was assessed according to Tanner staging (16). A radiograph of the left hand was used to determine BA using the Greulich-Pyle method (17). This assessment was made in a blinded fashion by a single pediatric endocrinologist. BA was defined as advanced when greater than the subject’s chronological age by one year or more (18).

Blood samples were drawn after an overnight fast for at least 12 hours at 8.00-10.00 a.m. before the first dose of steroids for assessment of serum levels of total cholesterol (TC), triglycerides (TG), high density lipoprotein-cholesterol (HDL-C), low density lipoprotein cholesterol (LDL-C), glucose and insulin. Serum TG and TC were assessed by quantitative enzymatic colorimetric technique (Bio Merieux-Diagnostic Chemicals Ltd., Charlottetown, CA, USA). Serum high-density lipoproteins (HDL) were measured by the phosphotungstate precipitation method (Biomerieux kit, Marcy L’etoile, Craponne, France). LDL cholesterol was calculated by Friedewald’s formula: (TC) - (HDL-C) - 1/5 (TG) (19). IR was calculated using the homeostasis model assessment for IR (HOMA-IR) equation formula: 

HOMA-IR=Fasting insulin (uU/mL) multiplied by fasting glucose (mmol/L) divided by 22.5. 

A cut-off level of 2.7 was used for diagnosing IR as previously described (20). ACTH, plasma 17-OHP, serum cortisol, androstenedione and testosterone were also measured with commercially available RIA kits (Siemens Healthcare Diagnostics Inc., Los Angeles, CA, USA). The plasma level of high sensitivity C-reactive protein (hsCRP) was measured using the hsCRP enzyme immunoassay test (ELISA) kit for quantitative determination of the CRP concentration in human serum (catalog no. E29-056; Immunospec Corp., Canoga Park, CA, USA).

### Echocardiographic Examination

All echocardiographic examinations were performed according to the recommendations of the American Society of Echocardiography (21). A Philips Envisor Ultrasound System with a S4-2 Broadband Sector (Philips Medical Systems, Inc., Netherlands) was used. Measurements were performed using the machine’s incorporated analysis package. An M-mode echocardiography was obtained at the left sternal border. Left ventricular (LV) dimension, LV fractional shortening (FS) and LV ejection fraction (EF) were measured. LV mass index (LVMI) was measured using a LVMI calculator. LV diastolic function was evaluated by mitral inflow velocities obtained in the apical four-chamber view. Mitral filling was assessed with the peak velocity of the transmitral early filling wave (E) and the peak velocity of atrial late filling (A) and the ratio of both (E/A) was calculated. The interval from the early peak velocity to the zero intercept of the extrapolated deceleration time (DcT) slope (early filling mitral DcT) was measured. The interval between the end of the LV outflow velocity and the onset of mitral inflow [isovolumic relaxation time (IVRT)] obtained by pulsed-wave Doppler with the cursor placed in the LV out-flow near the anterior leaflet of the mitral valve, was measured from the end of the LV ejection to the onset of the mitral inflow.

### Epicardial Fat Thickness Measurement

**Figure 1:**
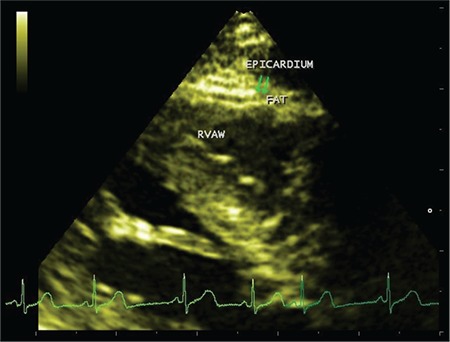
Echocardiographic imaging of the epicardial fat

A two-dimensional (2D) echocardiogram, using a standardised procedure, was performed with the patient in the left lateral decubitus position. EFT thickness was measured by an experienced pediatric echocardiologist, who was blinded to the subjects’ clinical and demographic data, using the procedure validated by Iacobellis et al (9). EFT was identified as the echolucent region between the external wall of the myocardium and the visceral layer of the pericardium (Figure 1). This thickness was measured perpendicularly on the free wall of the right ventricle at the end of systole over three cardiac cycles, using a parasternal long and a parasternal short axis. The average value of the three cardiac cycles from each echocardiographic view was used for the statistical analysis.

### Carotid Intima Media Thickness Measurement

All participants underwent an ultrasound scan to measure CA-IMT. The studies were performed in the morning between 7:30 and 9:30 a.m. after the children had fasted overnight. All ultrasound scans were performed by an experienced vascular operator who was unaware of the subject’s clinical details. Examination of CA-IMT was manually performed using a color duplex flow imaging system (Acuson 128 XP; Acuson Corporation, Mountain View, CA, USA). The examinations were performed while the patients were in a supine position, with their necks slightly extended and their heads turned 450 away from the examination side. From both sides of the head, three images were obtained from the distal common carotid artery, 1-2 cm proximal to the carotid bulb at end diastole. These images were then stored for later offline analyses. All studies were done according to a predetermined, standardized scanning protocol for the right and left carotid arteries (22). All measurements were performed in all participants by the same pediatric cardiologist who was blinded to the clinical and treatment status of the study participants. Reliability of echocardiographic measurements of CA-IMT and EFT were assessed by intra-observer correlation coefficient in all subjects.

### Statistical Analysis

Statistical analysis was performed using the Statistical Package for Social Sciences (SPSS) for Windows, version 16.0 (SPSS Inc, Chicago, IL, USA). Data were expressed as means + standard deviation. Comparisons of quantitative variables between the study groups were made using the paired Student t-test. Correlations between EFT and demographic, clinical, and laboratory variables were assessed using Pearson test. Multiple logistic regression analysis was used to determine the factors that were significantly associated with high EFT. The odds ratios, 95% confidence intervals and significances were calculated. For all tests, values of p<0.05 were considered statistically significant.

## Results

Demographic and anthropometric data of the patients and controls are shown in Table 1. Bone age in the patient group was advanced by an average of two years compared with chronological age. Compared with healthy controls, children with CAH exhibited increased visceral adiposity, as suggested by higher values of BMI SDS, waist circumference, hip circumference and waist to height ratio. Moreover, CAH children had higher SBP and DBP, although all children had blood pressures within the normal ranges. 

Laboratory data of the patients and controls are shown in Table 2. Concentrations of TC, TG, LDL-C, fasting blood glucose, fasting insulin, hsCRP, 17-OHP, androstenedione and testosterone were significantly higher while concentration of HDL-C was significantly lower in CAH patients compared to control subjects. HOMA-IR values were also significantly higher in the patients compared with controls.

The results of echocardiographic EFT and CA-IMT examinations are shown in Table 3. There were no significant differences in EF and FS values between patients and control subjects. However, compared to control subjects, patients had higher LVMI value, indicating myocardial hypertrophy, and lower E/A ratio, higher IVRT values and prolonged mitral DcT, indicating impaired diastolic function and increased CA-IMT and higher EFT. Intra-observer agreement on CA-IMT and EFT measurements were excellent. Intra-observer correlation coefficient was 0.94 and 0.95, respectively, indicating excellent reproducibility of these measures. Compared to patients who were well controlled (n=16), patients who were uncontrolled (n=20) were older, had advanced bone ages and had higher levels of 17-OHP, testosterone and hsCRP. In addition poorly controlled patients had higher values of LVMI, mitral DcT, EFT and CA-IMT (see Table 4). 

EFT thickness showed a statistically significant positive correlation with BMI, waist circumference, SBP, DBP, HOMA-IR, hsCRP, 17-OHP, testosterone, LVMI, mitral DcT and CA-IMT (see Table 5).

Multivariate analysis in children with CAH revealed that EFT was significantly correlated with waist circumference, 17-OHP, HOMA-IR, testosterone, mitral DcT, CA-IMT and LVMI (see Table 6).

## Discussion

This study demonstrates that a) children with classical CAH may have subclinical LV hypertrophy, diastolic dysfunction and subclinical atherosclerosis; b) EFT was higher in patients with CAH than in the healthy controls; c) EFT is correlated to carotid intima media thickness, LV mass and mitral DcT suggesting that EFT may be used as an additional marker of endothelial and myocardial dysfunction in children with CAH. The classical cardiovascular risk factors in children with CAH, namely obesity, hypertension, dyslipidemia, steroid treatment and others have been extensively discussed elsewhere (4,5,6).

To our knowledge, this study is the first to demonstrate that EFT was significantly increased in children with classical CAH compared with control children (p<0.001). In addition, we showed that EFT was correlated positively with CA-IMT. The multiple linear regression analysis showed that the CA-IMT was the variable that most influenced EFT. EFT was reported to be increased in children with a positive family history of type 2 DM and has been suggested as a risk factor for early atherosclerosis (23). A meta-analysis showed that EFT may be an effective marker for the prediction of coronary heart disease (24). Epicardial fat is thought to play a pivotal role in the pathogenesis of coronary artery disease (CAD) as it releases a wide range of biologically active molecules that modulate vascular smooth-muscle contraction (25). The paracrine effects of these molecules might be attributable to their location being close to the adventitia and extravascular bed (26). Gastaldelli and Basta (27) reported the existence of a link between epicardial fat and hypertension, atherosclerosis and coronary heart disease. Several studies have emphasized the link between EFT and the severity of CAD (28). Epicardial fat has an important role in the inflammatory process within the atherosclerotic plaque (29). Furthermore, it has been shown that epicardial fat products induce increased cell surface expression of adhesion molecules, enhance adhesion of monocytes to coronary artery endothelial cells, and facilitate migration of adherent monocytes (30).

Echocardiographic EFT measurements, provide some advantages when used to assess the cardiometabolic risk. They are objective, quantified, non-invasive, low cost, have routine applicability, avoid exposure to radiation and have a potential for monitoring therapeutic effects. They may also be used as a simple marker for identification of CAH patients with higher cardiovascular risk who may need further cardiac evaluation (31). 

In the present study, children with CAH had echocardiographic changes indicating the presence of LV hypertrophy, as indicated by increased LVMI. Moreover, our study showed significant positive correlations between EFT and LVMI that remained significant after regression analysis, which suggested a detrimental effect of EFT excess on the myocardium of patients with CAH. This is in agreement with Corradi et al (32) who reported that EFT levels have an important role in LV hypertrophy. Some mechanisms may be suggested to explain this correlation. It could be assumed that the increased visceral fat directly effects LV output and stroke volume to perfuse the increased body mass. Additionally, the biochemical properties of visceral adipose tissue, such as increased IR, high free fatty acids (FFA) levels, and adrenergic activity, could contribute to LV hypertrophy (33).

In the present study, children with CAH had echocardiographic changes indicating presence of diastolic dysfunction (as evidenced by reduced E/A ratio and prolonged IVRT and mitral DcT). Regression analysis revealed that EFT in CAH patients correlates with mitral DcT. This is in agreement with the study of Van der Meer (34) which showed that myocardial fat has progressive and harmful effects on LV diastolic function. Diastolic dysfunction has been considered as one of the first echocardiographic abnormalities to appear in patients with atherosclerotic cardiovascular disease with a high rate of release of FFA (35), which encounter no physical barrier or fascia before reaching the cardiomyocytes (36). Therefore, the myocardium receives a double dose of FFA from both the epicardial fat and the systemic circulation. Epicardial fat is a source of several bioactive molecules that might directly influence the myocardium (37). In metabolic and cardiovascular disease states, these fat tissues expand, becoming hypoxic and dysfunctional and recruiting phagocytic cells which would lead to a reduction in the production of protective cytokines and, eventually, impaired cardiac function (38,39).

BMI and waist circumference are widely accepted measures of generalized adiposity. However they are poor indicators for visceral obesity. It is well known that visceral adipose tissue accumulation is associated with subclinical atherosclerosis and increased cardiovascular mortality and morbidity. In this study, we found a very good correlation between EFT and waist circumference by multiple regression analysis in children with CAH. However we did not find a significant correlation between BMI-SDS and EFT. These findings suggest that waist circumference is a better anthropometric cardiovascular risk predictor and support the evidence that EFT is related to visceral fat, rather than total adiposity (40). Mavri et al (41) suggested that CA-IMT regression may also be achieved by weight reduction programs. Altin et al (42) suggested that laparoscopic sleeve gastrectomy induced weight loss results in regression of CA-IMT and EFT. Marked adipose mass reduction is associated with dramatic changes in circulating adipokine levels, with leptin reduction and adiponectin increase, thereby leading to a reduced leptin/adiponectin ratio. Of note, such a ratio was found to be directly correlated with CA-IMT in male subjects (43).

Testosterone concentrations were significantly higher in our subjects with CAH compared to controls, particularly in children who were poorly controlled on medical treatment. In addition, testosterone correlated positively and significantly with EFT. Colgecen et al (44) reported that subjects in advanced stages of androgenetic alopecia had higher echocardiographically measured EFT than controls. Moreover, Cakir et al (45) reported a strong positive correlation between testosterone concentrations and EFT in patients with polycystic ovarian disease. This finding suggests that androgen excess may be responsible for the increased EFT in patients with CAH. Physicians treating these patients should be aware that amelioration of androgen excess in patients with CAH should also be considered as a way to prevent cardiovascular symptoms and not only as a tool to improve hyperandrogenic symptoms.

In the present study, children with CAH had higher HOMA-IR than controls. EFT correlated positively and significantly with HOMA-IR by multiple regression analysis. This finding is in agreement with Manco et al (46) who reported that epicardial fat is a significant marker of increased insulin resistance. These observations suggest that epicardial fat is a tissue with high IR (47). EFT is associated with high lipolytic activity, probably because of the reduced antilipolytic effect of insulin in this tissue and an increased expression of B-adrenergic receptors, especially B-3 receptors. Stimulation by these receptors activates lipolysis and increases the release of FFAs which are able to promote blood pressure increase through different pathways, including adrenergic stimulation, increased oxidative stress, endothelial dysfunction and vascular cell growth (48).

### Study Limitations

We recognize that this study has some limitations such us; Due to the difficulties in enrolling and studying CAH children, the sample was relatively small in size and included patients with a wide age range. Due to the cross-sectional design, it is difficult to generalize the results to the general population. CA-IMT measurements were performed manually. We were not able to confirm EFT using the standard magnetic resonance imaging methods. However, echocardiographic calculation of epicardial fat has been reported to show good reliability when compared with magnetic resonance epicardial fat measurements (49). Epicardial adipose tissue has a three-dimensional distribution. Therefore 2D echocardiography may not accurately reflect the total amount of epicardial adiposity.

## Conclusions

EFT is increased in children with classic CAH, particularly in those with poor control and is correlated with CA-IMT, LV mass and mitral DcT. Measurement of EFT by echocardiography in CAH children may help to identify those at high risk of developing LV dysfunction and subclinical atherosclerosis. Future prospective and multicenter studies are required to confirm our results.

## Figures and Tables

**Table 1:**
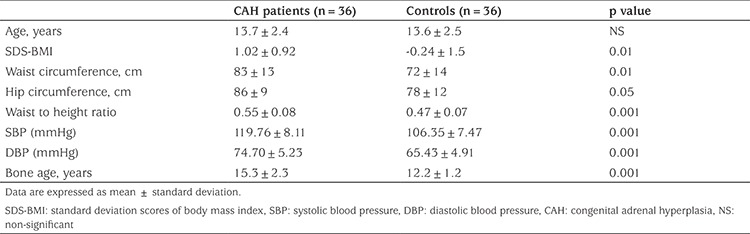
Demographic, anthropometric and clinical data of the congenital adrenal hyperplasia patients compared with controls

**Table 2:**
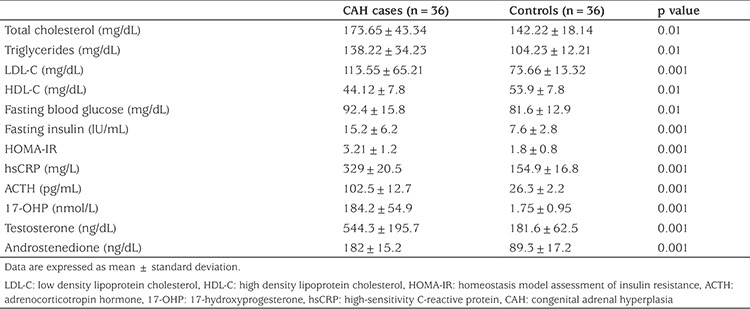
Laboratory data of the congenital adrenal hyperplasia patients compared with controls

**Table 3:**
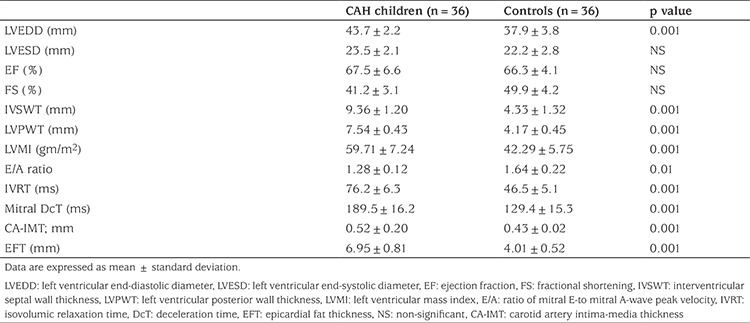
The echocardiographic, carotid artery intima-media thickness and epicardial fat thickness findings in the congenital adrenal hyperplasia patients compared to controls

**Table 4:**
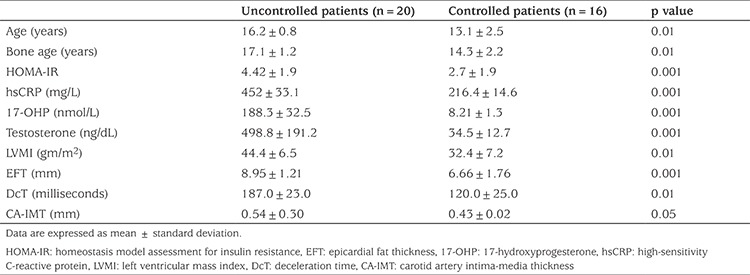
The demographic, laboratory and echocardiographic characteristics of patients, according to the degree of control on medical treatment

**Table 5:**
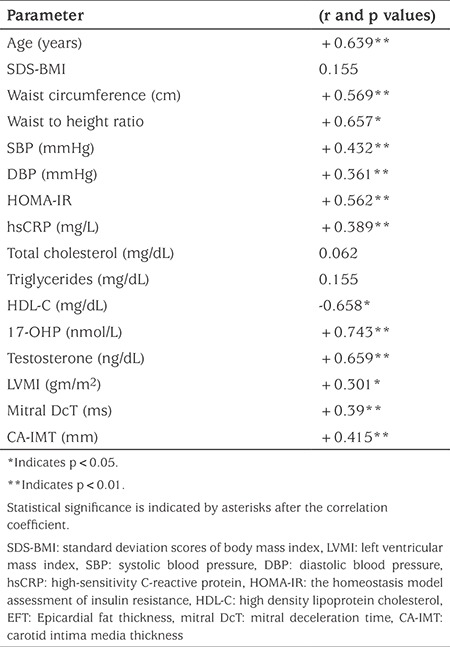
The correlation between epicardial fat thickness and anthropometric, laboratory and echocardiographic data in children with congenital adrenal hyperplasia

**Table 6:**
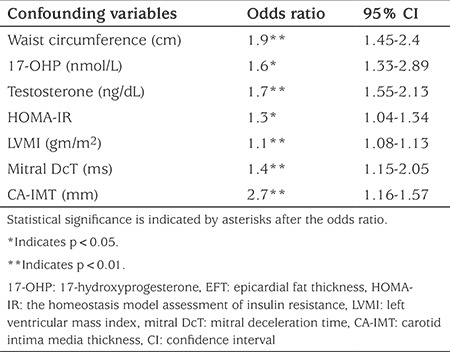
Multivariate correlation coefficients between epicardial fat thickness and various confounding variables in children with congenital adrenal hyperplasia
